# Supplementary material for: Human Cancer-Associated Mutations of SF3B1 Lead to a Splicing Modification of Its Own RNA
Source: Cancers (Basel). 2020 Mar 11;12(3):652. doi: 10.3390/cancers12030652 (PMC7139785; doi:10.3390/cancers12030652)
Supplement: Supplementary file 1 [file cancers-12-00652-s001.pdf]

# Human cancer-associated mutations of SF3B1 lead to a splicing modification of its own RNA

**Tiffany Bergot[1], Eric Lippert[1,2,3], Nathalie Douet-Guilbert[1,2,3,4], Séverine Commet[1], Laurent Corcos[1] and Delphine Bernard[1,2,*]**

[1] Univ Brest, Inserm, EFS, UMR1078, GGB, F-29200 Brest, France
[2] Centre de Ressources Biologiques du CHRU de Brest, France
[3] Hématologie Biologique, CHRU de Brest, France
[4] Génétique, CHRU de Brest, France

## Supplementary Materials and Methods

**Immunofluorescence**

After 48h of doxycycline induction, 300 000 cells were cultured on a coverslip coated with poly-L-lysine and incubated overnight in RPMI 1640 medium (Gibco), supplemented with 10% fetal bovine serum, 2 mM of L-glutamine and with 2 μg/mL of doxycycline. Cells were fixed with 4% para-formaldehyde and permeabilized with a PBS, 0,2% triton solution. Following incubation with primary anti-FLAG antibodies (1:1000, #F3165, Sigma) and secondary antibodies (1:1000, A-21121, Invitrogen), assembly between slide and cover-slide was done with the liquid mountant SlowFade™ Gold Antifade Mountant with DAPI (Invitrogen).

## Supplementary figure legends

**Table S1.** Features of the MDS patients included in this study.

**Table S2.** Primers used in this study.

**Figure S1**. Complementary results obtained in K562 and UT-7 cells upon inducible expression of SF3B1$^{K700E}$ or upon transitory expression of other important SF3B1 variants. (**A**) - Detection of SF3B1 proteins in K562 cells following inducible expression of *SF3B1*$^{WT}$ or *SF3B1*$^{K700E}$. The immunoblot shows three independent K562 cell populations expressing *SF3B1*$^{WT}$ (left) or *SF3B1*$^{K700E}$ (right) (I: induced, NI: non- induced). (**B**) Detection of SF3B1 proteins in K562 expressing distinct *SF3B1* variants. K562 cells were transiently transfected by plasmids expressing *SF3B1*$^{WT}$, *SF3B1*$^{K700E}$, *SF3B1*$^{E622D}$, *SF3B1*$^{H662Q}$ and *SF3B1*$^{K666E}$. (**C**) Detection of SF3B1 proteins in UT-7 cells following inducible expression of *SF3B1*$^{WT}$ or *SF3B1*$^{K700E}$. The immunoblot shows two independent UT-7 cell populations expressing *SF3B1*$^{WT}$ (left) or *SF3B1*$^{K700E}$ (right) (I: induced, NI: non-induced). In (A-C), total SF3B1 proteins (endogenous and exogenous) were detected using anti-SF3B1 antibody. Plasmid-encoded SF3B1 was detected using anti-FLAG antibody.

**Figure S2**. SF3B1ins$_{FLAG}$ proteins are correctly addressed to the nucleus. Immunofluorescence staining of SF3B1-FLAG proteins in inducible K562 cell lines expressing various SF3B1 constructs. Nuclei were stained with DAPI.

**Figure S3**. Splicing analysis of RBM5, DUSP11, CCNA2 and STK6 in inducible K562 cell lines expressing shSF3B1 alone or in combination with various *SF3B1* constructs. (**A**) Analysis of splicing events known to be specifically altered upon SF3B1 loss of function (RBM5, DUSP11) or *SF3B1*$^{mut}$ background (ENOSF1, TMEM14C, DPH5). (**B**) Quantification of CCNA2 or STK6 exon skipping was done by RT-qPCR, using primers allowing specific quantification of exon skipping events in CCNA2 (exon 5) and STK6 (exons 4, 5 and 6) transcripts, normalized to GAPDH.

**Figure S4**. Expression of the main disease-related *SF3B1* mutations does not affect growth of the *Schizosaccharomyces pombe prp10-1* strain. (**A**) Multiple alignment of the predominantly affected SF3B1 protein region in MDS (ClustalW software). The mouse (MOUSE), human (HUMAN), *Schizosaccharomyces pombe* (SCHPO) and *Saccharomyces cerevisiae* (YEAST) sequences are presented. The amino acids substitutions E622, R625, K666 and K700 found specifically in myelodysplastic syndromes are indicated in color. The lysine K700 residue, the most commonly affected in MDS, is preserved in *S. pombe* but not in *S. cerevisiae*. (**B**) *prp10-1* mutant cells were transformed with the empty vector pREP41, or a pREP41-HA expressing the wild type or mutated alleles of Sp*SAP155*, namely the counterparts of E622N, H662Q, K666N (R666N) and K700E. Streaks from independent transformants were made on EMM 2% glucose, replica-plated on EMM 2% glucose and cultured at 26°C or 37°C. (**C**) RT-PCR splicing analysis of TFIID and RPL7 transcripts in the *prp10-1* strain expressing different versions of Sp*SAP155*. Transformants were cultured at 26°C and subjected to a temperature switch at 37°C for 2 hours. RNA was extracted and analyzed by RT-PCR using specific primers allowing the detection of both spliced and un-spliced mRNA.

**Figure S5**. Uncropped blots corresponding to Figure 2A and Figure 2C. Densitometric ratios of SF3B1 or SF3B1-FLAG to GAPDH or β-actin are indicated for each lane.

**Figure S6**. Uncropped blots corresponding to Figure S1A, Figure S1B and Figure S1C. Densitometric ratios of SF3B1 or SF3B1-FLAG to GAPDH or β-actin are indicated for each lane.

Supplementary figures
Bergot et al.

**Figure S1**

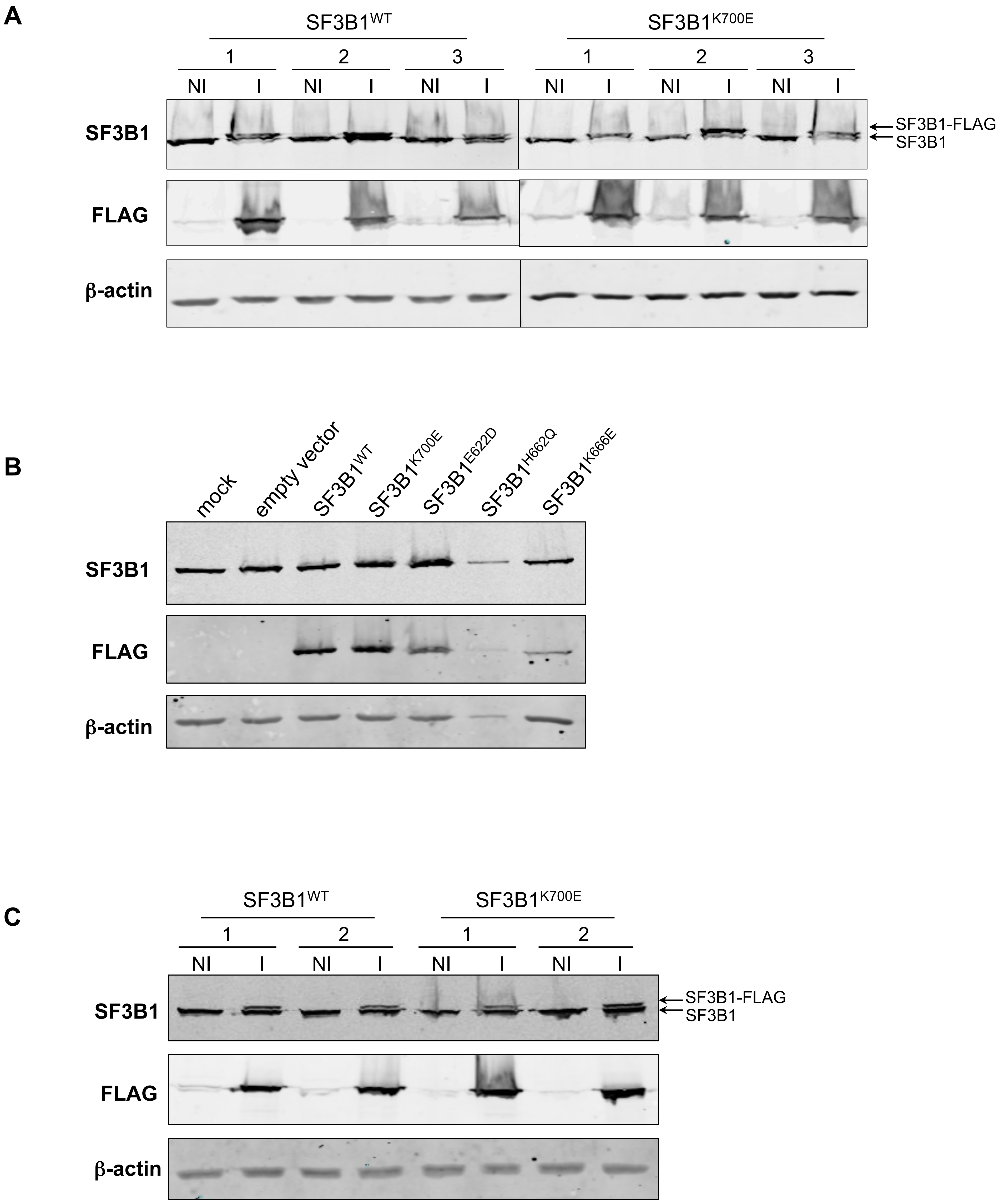
A
SF3B1WT
SF3B1K700E
1
2
3
NI
I
SF3B1
FLAG
β-actin
SF3B1-FLAG
SF3B1
B
mock
empty vector
SF3B1WT
SF3B1K700E
SF3B1E622D
SF3B1H662Q
SF3B1K666E
SF3B1
FLAG
β-actin
C
SF3B1WT
SF3B1K700E
1
2
NI
I
SF3B1
FLAG
β-actin
SF3B1-FLAG
SF3B1

Figure S2

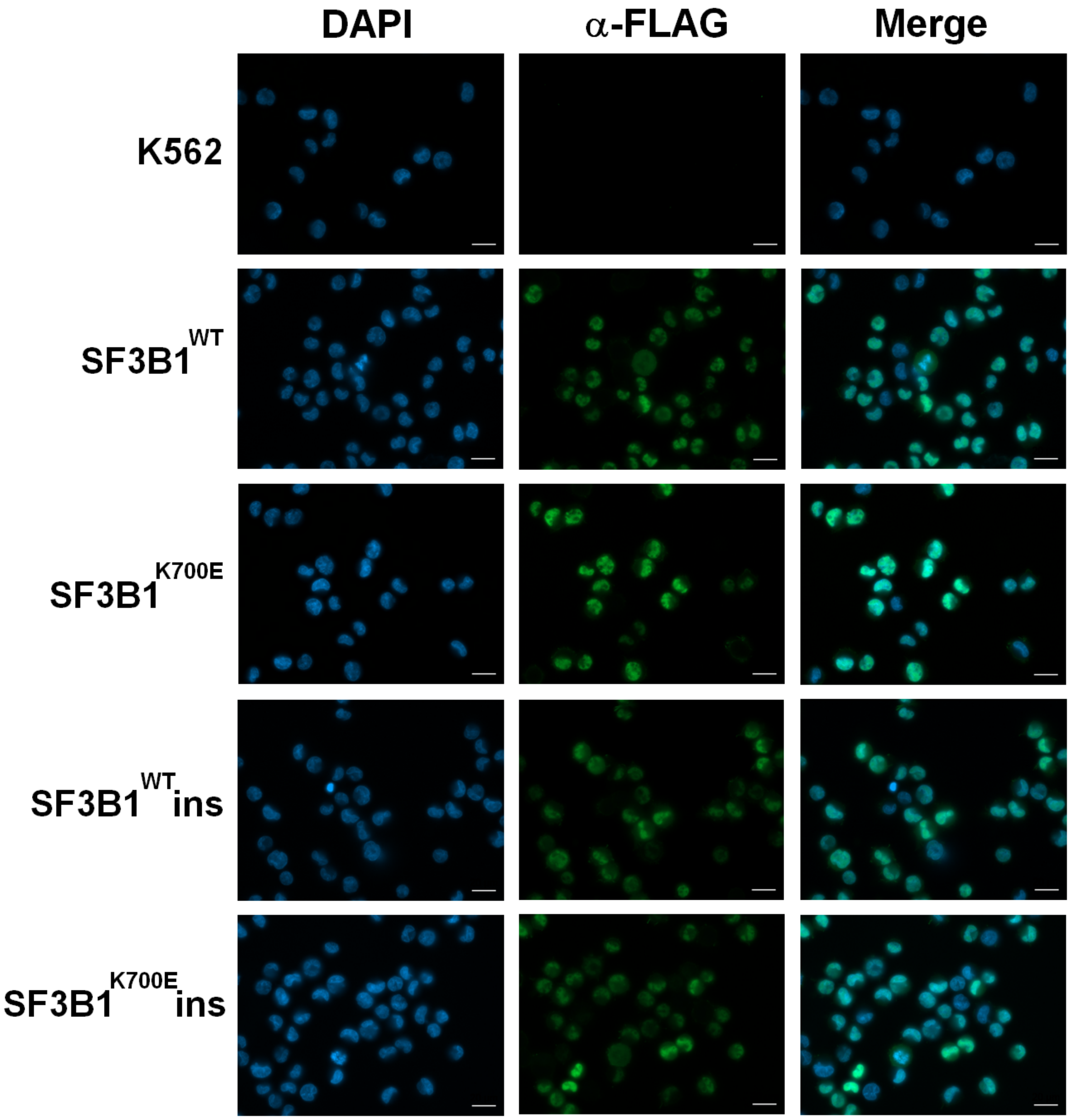
DAPI
α-FLAG
Merge
K562
SF3B1WT
SF3B1K700E
SF3B1WTins
SF3B1K700Eins

**Figure S3**

**A**

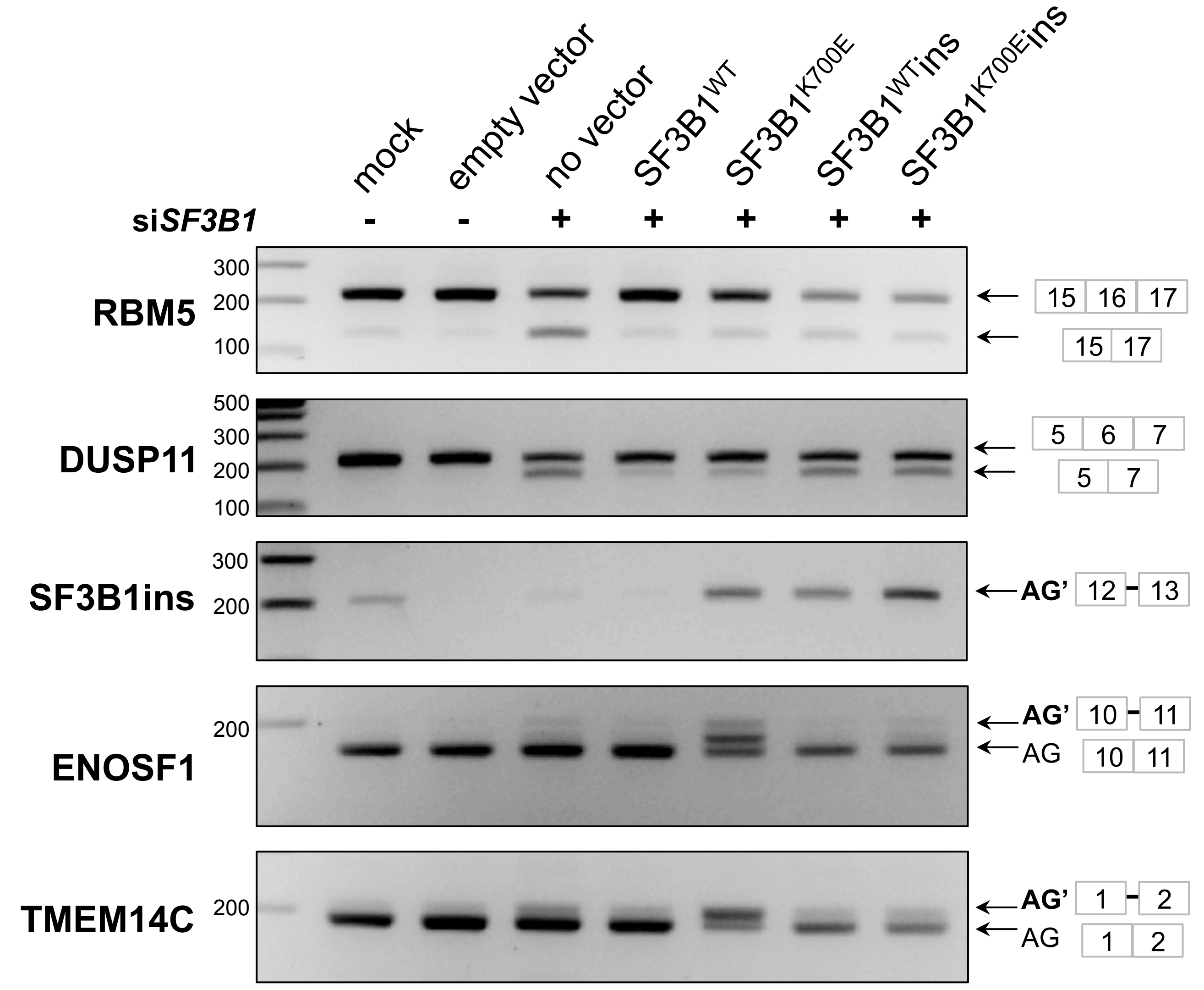
mock
empty vector
no vector
SF3B1WT
SF3B1K700E
SF3B1WTins
SF3B1K700Eins
siSF3B1
RBM5
DUSP11
SF3B1ins
ENOSF1
TMEM14C
300
200
100
500
AG'
AG
15 16 17
15 17
5 6 7
5 7
12 13
10 11
1 2

**B**

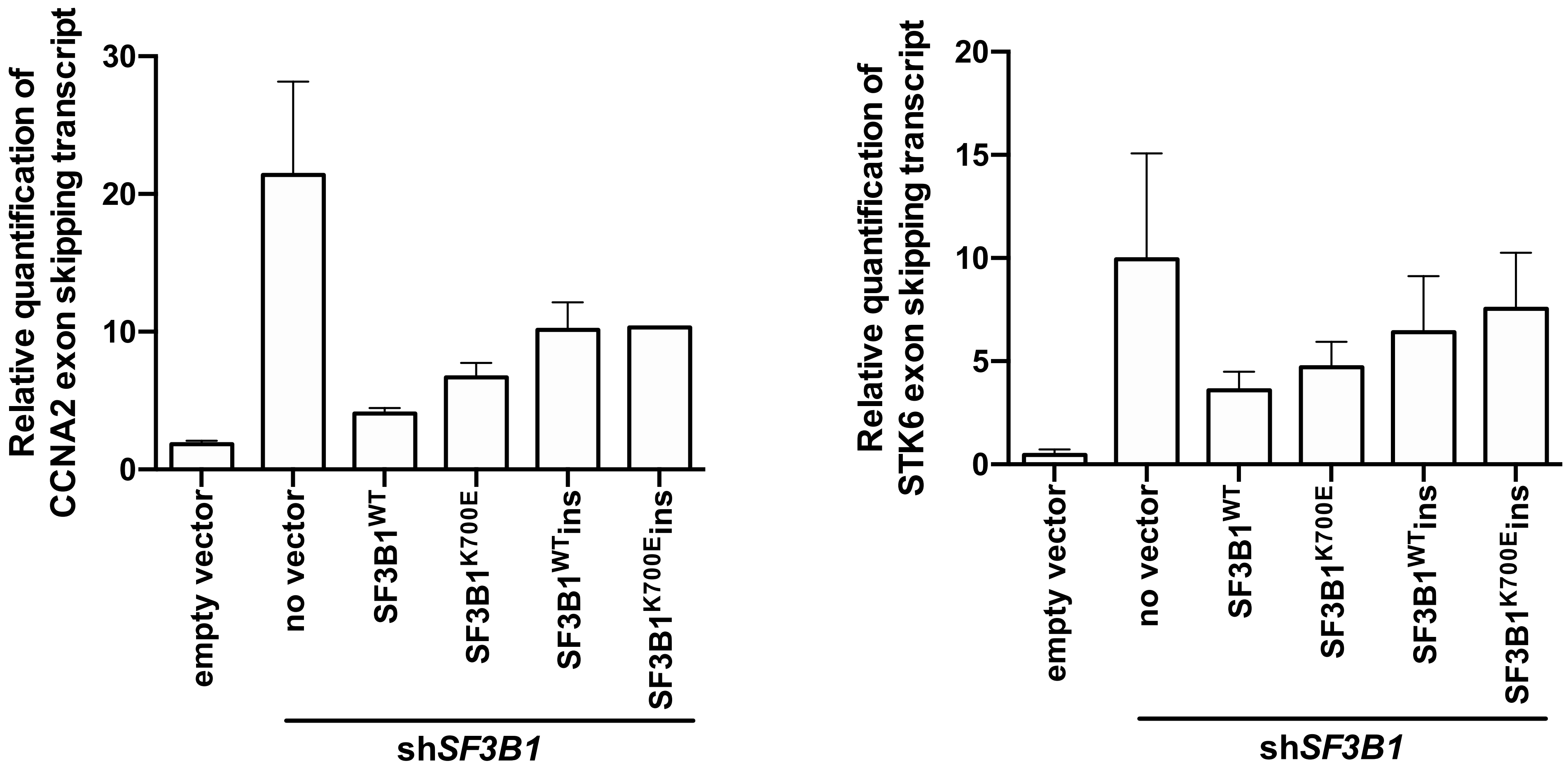
Relative quantification of CCNA2 exon skipping transcript
Relative quantification of STK6 exon skipping transcript
empty vector
no vector
SF3B1WT
SF3B1K700E
SF3B1WTins
SF3B1K700Eins
shSF3B1
0
10
20
30
5
15

# Figure S4

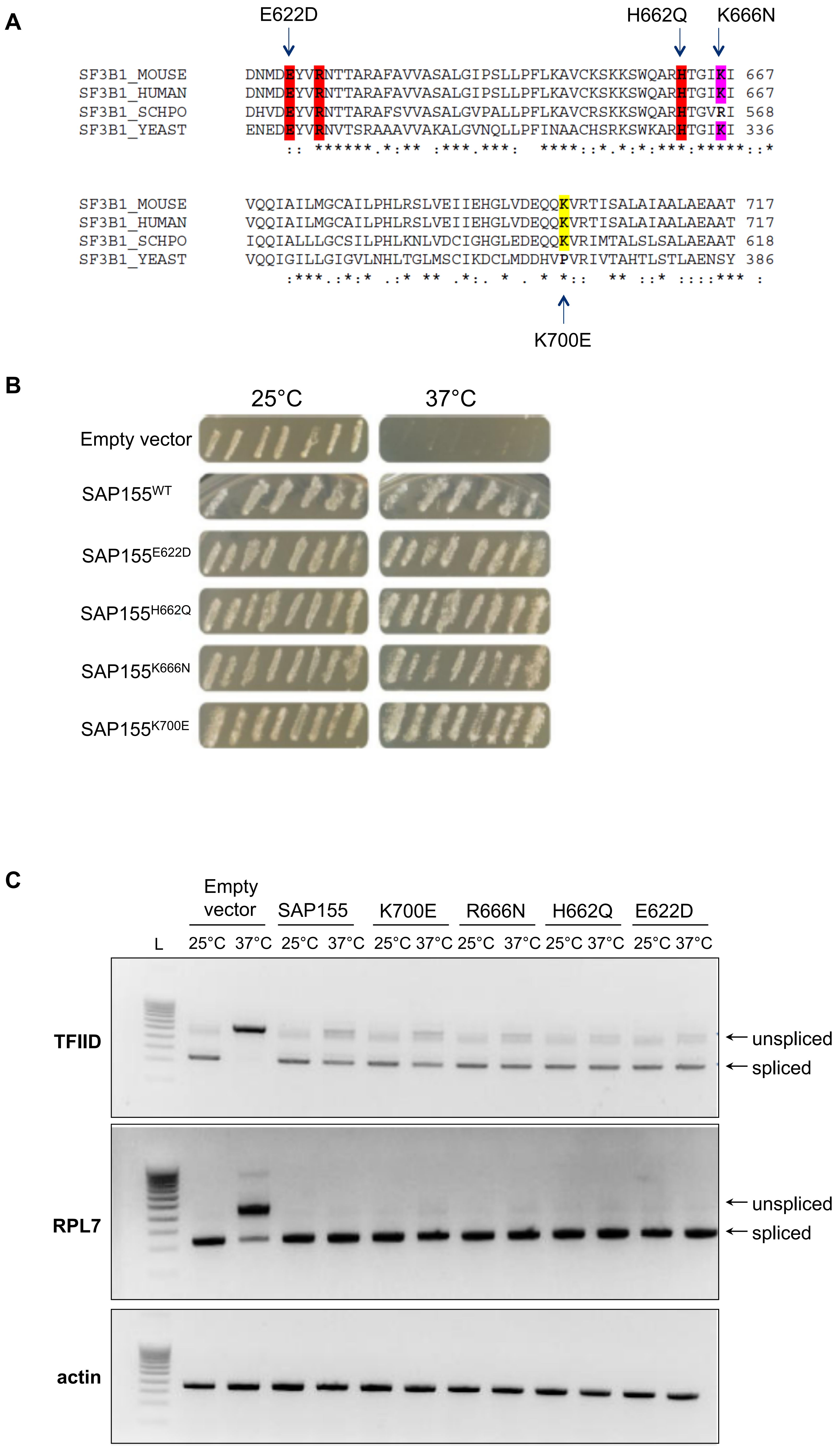
A
E622D
H662Q
K666N
SF3B1_MOUSE DNMDEYVRNTTARAFAVVASALGIPSLLPFLKAVCKSKKSWQARHTGIKI 667
SF3B1_HUMAN DNMDEYVRNTTARAFAVVASALGIPSLLPFLKAVCKSKKSWQARHTGIKI 667
SF3B1_SCHPO DHVDEYVRNTTARAFSVVASALGVPALLPFLKAVCRSKKSWQARHTGVRI 568
SF3B1_YEAST ENEDEYVRNVTSRAAAVVAKALGVNQLLPFINAACHSRKSWKARHTGIKI 336
SF3B1_MOUSE VQQIAILMGCAILPHLRSLVEIIEHGLVDEQQKVRTISALAIAALAEAAT 717
SF3B1_HUMAN VQQIAILMGCAILPHLRSLVEIIEHGLVDEQQKVRTISALAIAALAEAAT 717
SF3B1_SCHPO IQQIALLLGCSILPHLKNLVDCIGHGLEDEQQKVRIMTALSLSALAEAAT 618
SF3B1_YEAST VQQIGILLGIGVLNHLTGLMSCIKDCLMDDHVPVRIVTAHTLSTLAENSY 386
K700E
B
25°C
37°C
Empty vector
SAP155$^{WT}$
SAP155$^{E622D}$
SAP155$^{H662Q}$
SAP155$^{K666N}$
SAP155$^{K700E}$
C
Empty vector
SAP155
K700E
R666N
H662Q
E622D
L
25°C 37°C 25°C 37°C 25°C 37°C 25°C 37°C 25°C 37°C 25°C 37°C
TFIID
unspliced
spliced
RPL7
unspliced
spliced
actin

**Figure S5**

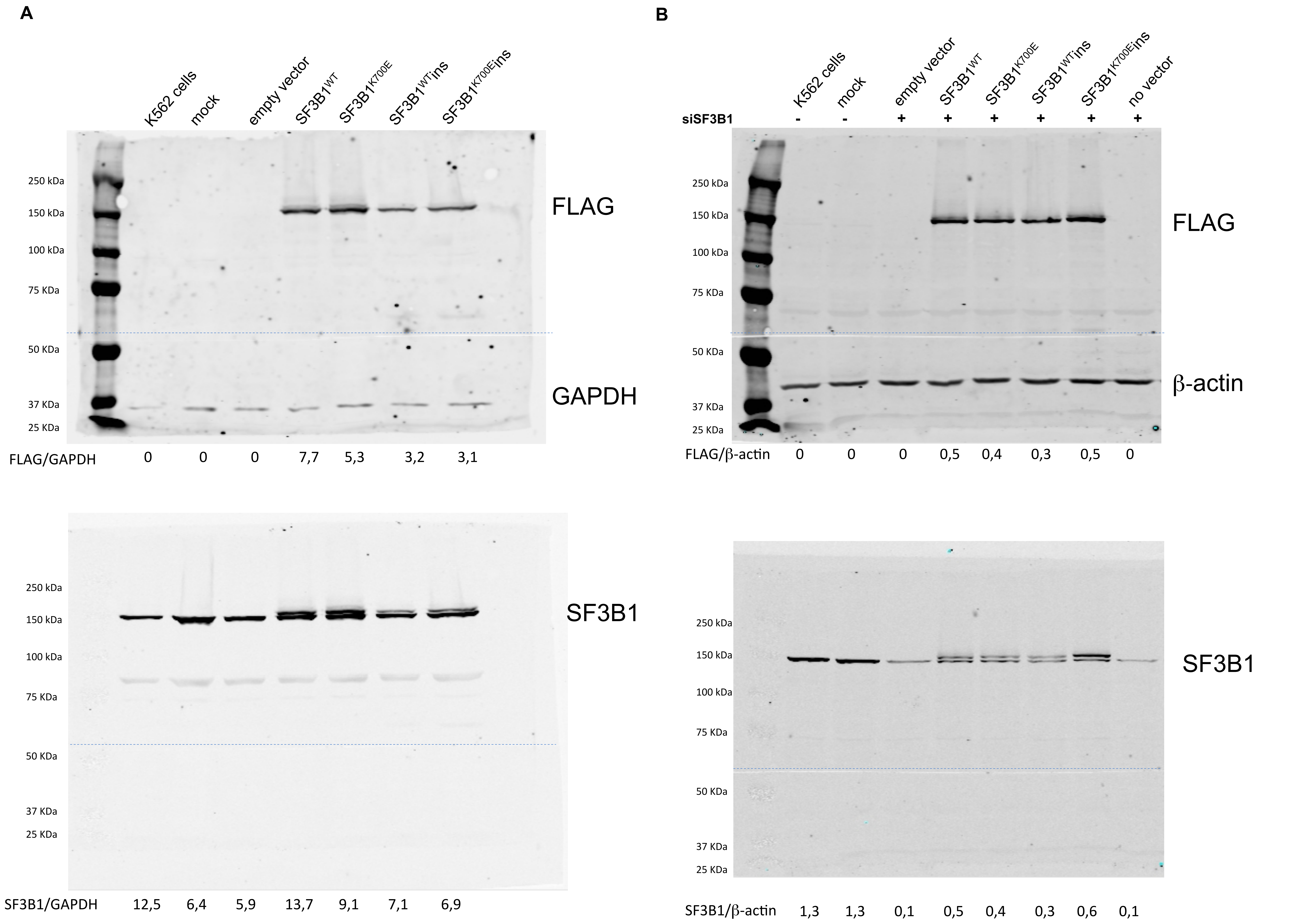


Membranes were cut to probe different antibodies (blue line)

**Figure S6**

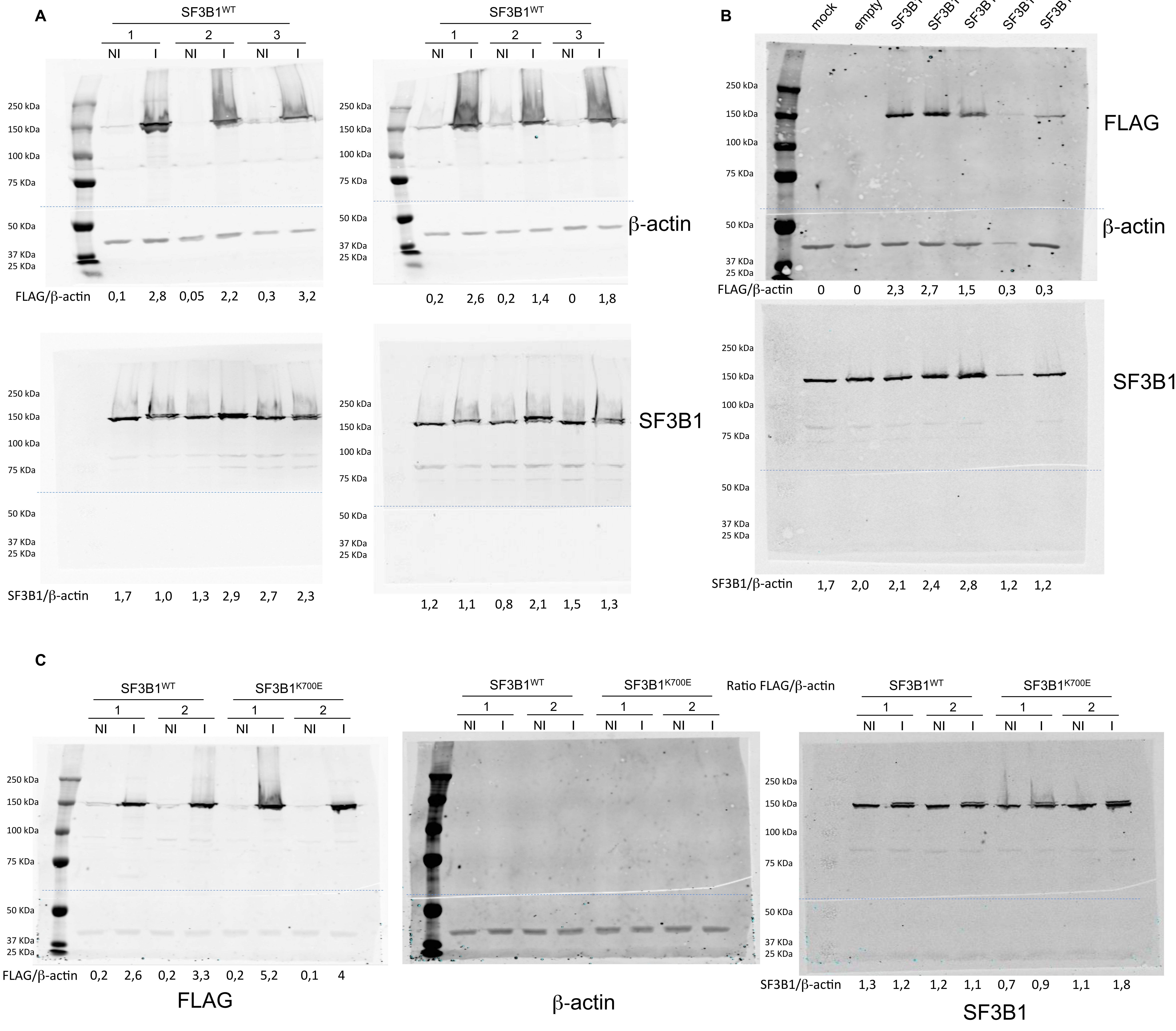


Membranes were cut to probe different antibodies (blue line)

# Supplemental Table S1

| N° | Blood features | Bone Marrow features | MDS classification according 2016 revision WHO classification | % RS | medullar karyotype | MDS Cytogenetic scoring system |
|---|---|---|---|---|---|---|
| 1 | cytopenia | No dysmyelopoiesis | ICUS | U | normal | |
| 2 | cytopenia | No dysmyelopoiesis | ICUS | U | normal | |
| 3 | cytopenia | No dysmyelopoiesis | ICUS | 0% | normal | |
| 4 | cytopenia | No dysmyelopoiesis | ICUS | 0% | normal | |
| 5 | anemia | Dysmyelopoiesis | MDS-SLD | 0% | normal | good prognosis |
| 6 | anemia | Dysmyelopoiesis | MDS-MLD | 0% | normal | good prognosis |
| 7 | anemia | Dysmyelopoiesis | MDS-MLD | 0% | normal | good prognosis |
| 8 | anemia | Dysmyelopoiesis | MDS-MLD | 0% | normal | good prognosis |
| 9 | anemia | Dysmyelopoiesis | MDS-MLD | 0% | normal | good prognosis |
| 10 | thrombopenia, neutropenia | Dysmyelopoiesis | MDS-MLD | 0% | normal | good prognosis |

| N° | Blood features | Bone Marrow features | MDS classification according 2016 revision WHO classification | % RS | medullar karyotype | MDS Cytogenetic scoring system | SF3B1 status (allele burden) | other mutated genes (NGS) |
|---|---|---|---|---|---|---|---|---|
| 1 | pancytopenia | Dysmyelopoiesis | MDS-EB1 | 44% | 48,XY,+8,+12[5]/46,XY[38] | intermediate prognosis | **wild type** | TET2, SRSF2, RUNX1 |
| 2 | thrombopenia | Dysmyelopoiesis | MDS-RS-MLD | 18% | normal | good prognosis | **wild type** | TET2, ZRSR2 |
| 3 | anemia | Dysmyelopoiesis | MDS-RS-MLD | 53% | 46,XY,del(20)(q11q13)[8]/45,X,-Y[8]/46,XY[7] | good prognosis | **R625C** (36,5%) R625L (7,3%) | TET2 |
| 4 | anemia | Dysmyelopoiesis | MDS-RS-MLD | 70% | normal | good prognosis | **K700E** (37%) | |
| 5 | anemia | Dysmyelopoiesis | MDS-RS-MLD | 15% | normal | good prognosis | **E622D** (41%) | |
| 6 | anemia, thrombopenia | Dysmyelopoiesis | MDS-RS-MLD | 62% | normal | good prognosis | **del gly740** (46%) | TET2, U2AF1, SH2B3 |
| 7 | anemia | Dysmyelopoiesis | MDS-RS-SLD | 12% | normal | good prognosis | **R625C** (33%) | TET2, EZH2, ASXL1 |
| 8 | anema | Dysmyelopoiesis | MDS-RS-SLD | 61% | normal | good prognosis | **K700E** (28,8%) | |
| 9 | anemia | Dysmyelopoiesis | MDS-RS-SLD | 53% | 45,X,-Y[12]/46,XY[9] | very good prognosis | **K666N** (12,3%) H662D (4,4%) | TP53 |
| 10 | anemia | Dysmyelopoiesis | MDS-RS-SLD | 70% | normal | good prognosis | **E622D** (40,35%) | |
| 11 | anemia | Dysmyelopoiesis | MDS-RS-SLD | 80% | normal | good prognosis | **K666N** (39%) | |
| 12 | anemia, thrombocytosis | Dysmyelopoiesis | MDS/MPN-RS- with Thrombocytosis | 90% | normal | good prognosis | **K700E** (48%) | MPL, DNMT3A, JAK2, ETNK1 |

ICUS : Idiopathic Cytopenia of Undetermined Significance
MDS-SLD : MDS wth single lineage dysplasia
MDS-MLD : MDS wth multilineage dysplasia
MDS-EB1: MDS with excess blasts-1
MPN: myeloproliferative neoplasms

# Supplemental Table S2

| | | Name gene | Primers sense | exon number | Sequences |
|---|---|---|---|---|---|
| PCR | *Homo sapiens* | SF3B1 | forward | exon 12 | GCTCGTGAATTTGGAGCTGG |
| | | | reverse | exon 13 | GCGGTTCAATGACCACGAGG |
| | | SF3B1ins | forward | exon 12 | GCATTGCGTCAGATTACTGA |
| | | | reverse | intron 12 | CTGAAAAAGAGAAAAGAGAAGAAG |
| | | DPH5 | forward | exon 4 | CTGTAGGCTGCTGTGGTTTAC |
| | | | reverse | exon 7-intron 7 | CCTTCCCCTATAGGCAGAAA |
| | | TMEM14C* | forward | exon 1 | GACACCTCGCAGTCATTCCT |
| | | | reverse | exon 3 | TGATCCCACCAGAAGCAACC |
| | | ENOSF1* | forward | exon 10 | GGGTGCTGATCTCCAGGATG |
| | | | reverse | exon 11 | GGAGGTTGGCTCCTCAATCC |
| | | RBM5 | forward | exon 15 | GGCTGTAGTGTCCCAGAGTC |
| | | | reverse | exon 17 | GCCCTGTTGTCGGATCATAG |
| | | DUSP11 | forward | exon 5 | GGACTGGCTACCTCATTTGC |
| | | | reverse | exon 8 | GACTGGTTGCATGAGATGTGC |
| | *S.pombe* | TFIID | forward | exon 1 | TTCCCTGTTCTCCCCAATGC |
| | | | reverse | exon 2 | ACTTGGGTTCACGGATACGC |
| | | NDA3 | forward | exon 5 | CGAGCAAATTCGCTCTGTTC |
| | | | reverse | exon 6 | CATTGCAGAAAACTGATCACC |
| | | RPL7 | forward | exon 1 | GTGTTGAGCAGGCTATTGCC |
| | | | reverse | exon 2 | GCGTAATAAGCGCAGAACTTTGCGAATTT |
| | | Actin | forward | / | GATTGTCGGTAGACCCCGTC |
| | | | reverse | / | CAGTCAACAAGCAAGGGTGC |
| qRT-PCR | *Homo sapiens* | CCNA2 | forward | exon 4-exon 6 | TGCTATGCTGTTAGCCTCTTTTT |
| | | | reverse | exon 6 | CCCGTGACTGTGTAGAGTGC |
| | | STK6 | forward | exon 3-exon 7 | CTGCCATCGGCACCTGTATAT |
| | | | reverse | exon 7 | GCATGTACTGACCACCCAAA |

* Primers from Dolatshad et al., 2016.

| | | Name | Primers sense | Application | Sequences |
|---|---|---|---|---|---|
| Cloning | *Homo sapiens* | SF3B1 E622D | forward | mutagenesis | CCAGATATTGACAACATGGACGATTACGTGAGGAATACCAC |
| | | | reverse | | GTGGTATTCCTCACGTAATCGTCCATGTTGTCAATATCTGG |
| | | SF3B1 H662Q | forward | | AAAGTTGGCAGGCCCGCCAGACCGGCATCAAGATTGTGC |
| | | | reverse | | GCACAATCTTGATGCCGGTCTGGCGGGCCTGCCAACTAA |
| | | SF3B1 K666E | forward | | GCCCGCCACACCGGCATCGAGATTGTGCAGCAGATCGCA |
| | | | reverse | | TGCGATCTGCTGCACAATCTCGATGCCGGTGTGGCGGGC |
| | | pCW57.1 | forward | Cloning | GTACAAACTTGTTGATGCTAGC |
| | | | reverse | | GGTTTAGTAATGAACCGGTC |
| | | SF3B1 | forward | | GCTAGCATCAACAAGTTTGTACCGCCACCATGGATTACAAG |
| | | | reverse | | ACCGGTTCATTACTAAACCCTACAGGATGTAGTCCAGTTCGTAAC |
| | | SF3B1ins | forward | | CTTCTTCTCTTTTCTCTTTTTCAGATCCTGGTGGTCATTGAGCC |
| | | | reverse | | CTTATGGACGTAGGGACGCAC |
| | *S.pombe* | SAP155 K700E | forward | mutagenesis | GAAGATGAACAACAAGAAGTACGAATTATGAC |
| | | | reverse | | GTACATAATTCGTACTTCTTGTTGTTCATCTTC |
| | | SAP155 R666N | forward | | CCAGGCATACTGGCGTTAACATTATTCAACAGATTGC |
| | | | reverse | | GCAATCTGTTGAATAATGTTAACGCCAGTATGCCTGG |
| | | SAP155 H662Q | forward | | CATGGCAAGCCAGGCAAACTGGCGTTCGGATTATTC |
| | | | reverse | | GAATAATCCGAACGCCAGTTTGCCTGGCTTGCCATC |
| | | SAP155 E662D | forward | | GATCATGTCGACGACTATGTCCGAAACACC |
| | | | reverse | | GGTGTTTCGGACATAGTCGTCGACATGATC |
